# Visceral condition assessment through digital tongue image analysis

**DOI:** 10.3389/frai.2024.1501184

**Published:** 2025-01-06

**Authors:** Siu Cheong Ho, Yiliang Chen, Yao Jie Xie, Wing-Fai Yeung, Shu-Cheng Chen, Jing Qin

**Affiliations:** School of Nursing, The Hong Kong Polytechnic University, Hong Kong, China

**Keywords:** tongue diagnosis, inspection of the tongue, Chinese medicine, five viscera, deep learning, multi-task learning

## Abstract

Traditional Chinese medicine (TCM) has long utilized tongue diagnosis as a crucial method for assessing internal visceral condition. This study aims to modernize this ancient practice by developing an automated system for analyzing tongue images in relation to the five organs, corresponding to the heart, liver, spleen, lung, and kidney—collectively known as the “five viscera” in TCM. We propose a novel tongue image partitioning algorithm that divides the tongue into four regions associated with these specific organs, according to TCM principles. These partitioned regions are then processed by our newly developed OrganNet, a specialized neural network designed to focus on organ-specific features. Our method simulates the TCM diagnostic process while leveraging modern machine learning techniques. To support this research, we have created a comprehensive tongue image dataset specifically tailored for these five visceral pattern assessment. Results demonstrate the effectiveness of our approach in accurately identifying correlations between tongue regions and visceral conditions. This study bridges TCM practices with contemporary technology, potentially enhancing diagnostic accuracy and efficiency in both TCM and modern medical contexts.

## 1 Introduction

Tongue diagnosis has been a cornerstone of Traditional Chinese Medicine (TCM) for millennia, offering crucial insights into a patient's overall health and internal organ conditions (Zhang et al., 2023; Foh et al., 2022; Chen, 1987; Dong et al., 2008; Shin et al., 2007). The color, shape, coating, and other characteristics of the tongue are believed to reflect the physiological and pathological states of different organs, particularly the five viscera—heart, liver, spleen, lung, and kidney (Solos et al., 2012; Zhao, 2019; Holroyde-Downing, 2017; Hui et al., 2007; Luiz et al., 2011). This concept of correspondence between tongue features and the five viscera is a fundamental principle in TCM diagnostics, as detailed in comprehensive Diagnostics of Traditional Chinese Medicine textbook on TCM diagnosis (Li, 2016).

With the rapid advancement of technology, particularly in the fields of machine learning and deep learning, there has been a growing interest in applying these computational methods to various aspects of TCM (Lv et al., 2023; Zheng et al., 2020; Li et al., 2020; Li and Yang, 2017; Cheng et al., 2021). These modern approaches offer the potential to enhance the objectivity, efficiency, and accuracy of traditional diagnostic techniques. However, previous research has primarily focused on tongue image partitioning and the identification of surface features such as cracks and tooth marks, rather than exploring the relationship between tongue characteristics and visceral condition. The challenge in identifying visceral condition through tongue images lies in the difficulty of associating tongue characteristics with specific organ conditions without the aid of prior knowledge. This connection is not immediately apparent and requires expertise in Traditional Chinese Medicine principles, making it a complex task for automated systems to learn and interpret accurately.

In this study, we propose a novel approach that diverges from previous tongue image studies. Our study only focus on the correlation of tongue inspection and the functions of five viscera (heart, lung, liver, spleen and kidney) at this stage. Through this approach, we aim to complement and expand the direction of tongue image research, offering new insights into the relationship between tongue characteristics and overall visceral condition.

**Figure 1:**
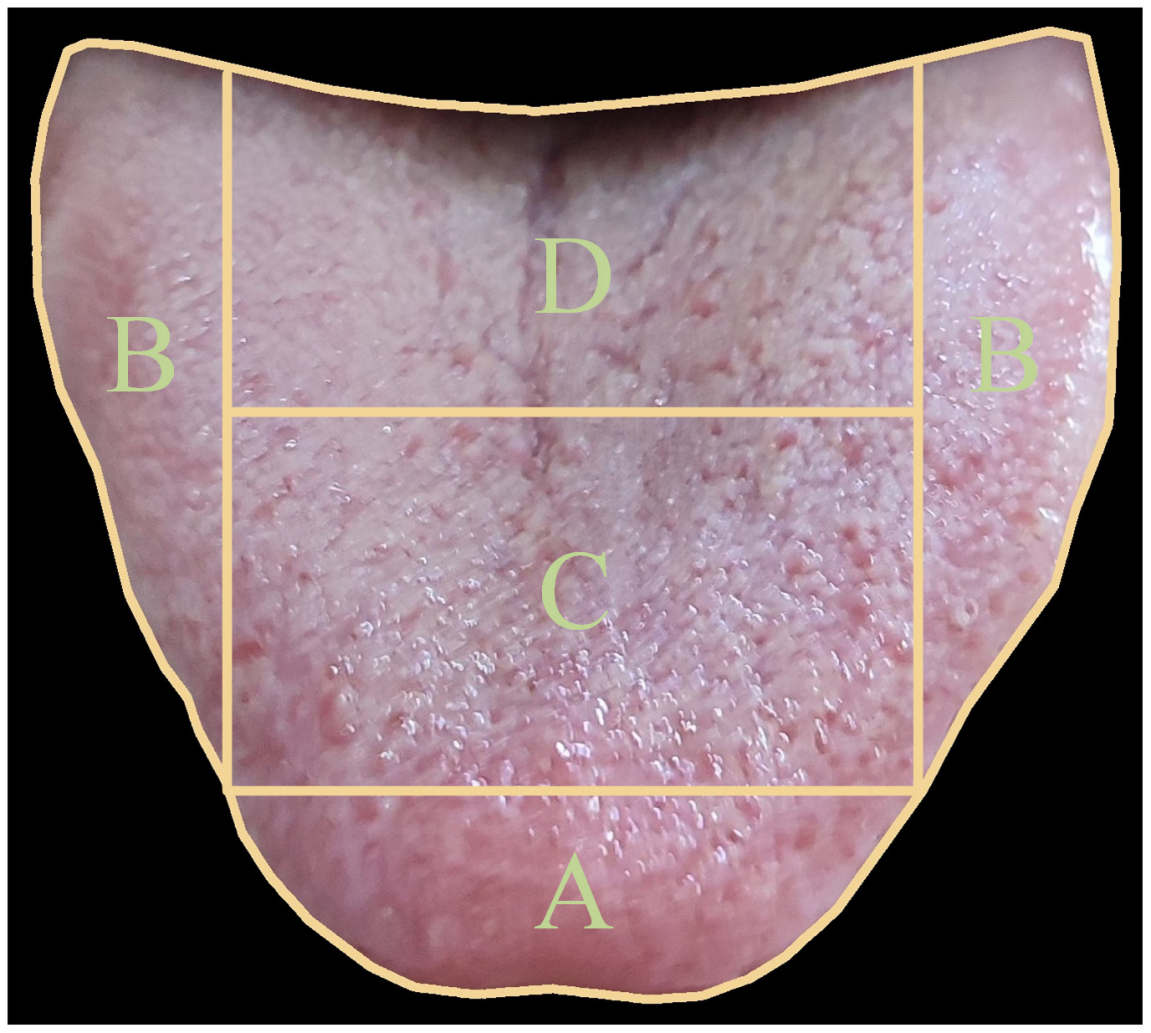
Tongue image partitioning for five viscera and bowel pattern identification in Traditional Chinese Medicine (TCM). The tongue is divided into four main regions: **(A)** The tip of the tongue, indicating heart and lung health; **(B)** The margins of the tongue, reflecting liver condition; **(C)** The center of the tongue, representing spleen health; and **(D)** The root of the tongue, corresponding to kidney function. This partitioning allows for a comprehensive analysis of viscera and bowel health based on TCM principles.

To achieve this goal, we have constructed an extensive dataset that establishes relationships between tongue images and the health status of five viscera. We have partitioned the tongue image into four regions (A, B, C, D) as shown in Figure 1, corresponding to specific viscera according to TCM principles documented in the comprehensive Diagnostics of Traditional Chinese Medicine textbook (Maciocia, 2015): (A) The tip of the tongue, indicating heart and lung health, providing insights into cardiovascular and respiratory conditions; (B) The margins of the tongue, reflecting liver condition, related to detoxification functions; (C) The center of the tongue, representing spleen health, which is essential for digestion and nutrient absorption; and (D) The root of the tongue, corresponding to kidney function, indicating the health of the renal system and the body's water balance. For example, a pale and swollen tip of the tongue might suggest issues such as heart qi deficiency or lung weakness, prompting further investigation and specific therapeutic approaches in TCM. Therefore, this partitioning allows us to focus on specific areas of the tongue that correspond to particular viscera, aligning with traditional TCM diagnostic methods. To leverage this partitioned approach, we have proposed a specially designed deep learning model called OrganNet. This network takes different tongue regions as input, allowing it to focus on the specific areas representing each viscera without interference from other regions. By doing so, OrganNet bridges the gap between real-world TCM diagnosis and deep learning, potentially improving the accuracy of visceral condition assessment based on tongue images.

Therefore, the main contributions of this study are threefold:

1. Construction of an extensive dataset of tongue images with corresponding visceral condition annotations.
2. Development of a tongue organ region partitioning algorithm and proposal of OrganNet, a novel network specifically designed for visceral condition recognition from tongue images.
3. Experimental results demonstrating performance surpassing advanced models and comparable to that of experienced TCM practitioners.

These contributions advance the application of computational techniques to traditional tongue diagnosis, potentially enhancing visceral condition assessment in clinical practice.

## 2 Related works

The field of tongue image analysis has witnessed a diverse array of computational approaches, ranging from traditional machine learning to advanced deep learning techniques.

Traditional machine learning methods have shown considerable promise in various aspects of tongue image analysis. Researchers have explored geometric feature extraction for automated tongue shape classification (Obafemi-Ajayi et al., 2012). Some studies have focused on specific tongue characteristics, such as cracks, by analyzing color components in different color spaces (Yang et al., 2010). Others have investigated the recognition of acantha and ecchymosis in tongue patterns through RGB color range and grayscale analysis (Xu et al., 2004). Efforts have also been made to extract prickles from green tongue images (Wang et al., 2016).

Several studies have applied machine learning to disease diagnosis based on tongue images. For instance, researchers have developed computer-based systems for diabetes detection by assessing visual variations on the tongue surface (Umadevi et al., 2019). Support vector machines (SVMs) have been employed alongside sophisticated image processing techniques to analyze tongue textures (Srividhya and Muthukumaravel, 2019). Some innovative approaches have utilized optimization algorithms for hyperglycemia diagnosis using large tongue image datasets (Naveed, 2022). A comprehensive study explored various machine learning algorithms and color space models for tongue disease prediction, addressing subjectivity issues in traditional diagnosis (Hassoon et al., 2024).

In recent years, deep learning methods have gained significant traction in tongue image analysis due to their powerful feature extraction capabilities. Deep residual neural networks have been utilized to identify tooth-marked tongues with high accuracy (Wang et al., 2020). CNN models combining U-NET and Discriminative Filter Learning have shown promising results in classifying different types of tongue coating (Xu et al., 2020). A prospective multicenter clinical study demonstrated the potential of deep learning in gastric cancer diagnosis using tongue images (Yuan et al., 2023).

The versatility of deep learning in analyzing complex tongue features has been showcased in studies on multi-label tongue image analysis (Jiang et al., 2022). Researchers have also developed lightweight architectures for real-time tongue image segmentation, achieving high accuracy across multiple datasets (Li et al., 2019). Advanced frameworks like improved Swin Transformers have been applied to recognize different stages of tongue tumor development, outperforming experienced specialists (Xu et al., 2024). Novel approaches combining techniques such as Vector Quantized Variational Autoencoder (VQ-VAE) and K-means clustering have been explored for tongue image classification in diabetes patients (Li et al., 2022).

In this research, we propose a novel approach that diverges from previous tongue image studies. Our focus is on utilizing tongue images to assess the health status of five major organs in the human body. Through this approach, we aim to complement and expand the direction of tongue image research, offering new insights into the relationship between tongue characteristics and overall organ health status.

## 3 Dataset

In this section, we describe the process of collecting our dataset, detail its structure, and outline the evaluation protocol used in our experiments.

### 3.1 Data collection

All images in our dataset were annotated on-site in clinical settings. Our data collection process involved a total of 4,645 participants, with one image captured for each individual. The resulting tongue images were pre-processed to ensure they were properly segmented, and we excluded any images that were obstructed or otherwise unsuitable for analysis.

The annotation process was meticulously conducted by three experienced TCM practitioners from Hong Kong. These practitioners reached a consensus on each label through a comprehensive diagnostic process that integrated multiple sources of patient data. The basis for each diagnosis and subsequent labeling included a detailed review of the patient's medical records, personal histories provided orally by the patients, and the practitioners' own assessments derived from direct, face-to-face consultations using the classical four diagnostic methods of TCM-inspection, auscultation and olfaction, inquiry, and palpation – to gather a holistic understanding of the patient's current and past health conditions. This rigorous approach ensured that the labeling was grounded in authentic TCM diagnostic practices, enhancing the reliability and validity of the study's data.

**Figure 2:**
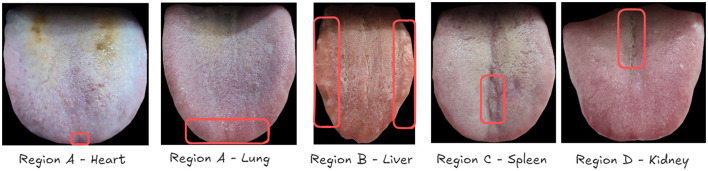
Examples of unhealthy organ annotations from our collected tongue image dataset. Each image is accompanied by health assessments for five viscera: heart **(A)**, lung **(A)**, liver **(B)**, spleen **(C)**, and kidney **(D)**, as annotated by TCM practitioners. The red circles indicate the areas where TCM practitioners identified features suggesting organ unhealthiness. These circles are for illustrative purposes in this example and do not exist in the actual annotations.

Our rigorous data collection and annotation process resulted in a high-quality dataset that accurately represents the diverse conditions encountered in clinical practice. Figure 2 showcases a selection of examples from our collected dataset, illustrating the variety of tongue images and their corresponding visceral condition annotations.

TCM practitioners assess the health status of different organs based on specific regions of the tongue. The liver, spleen, and kidney are evaluated using regions B, C, and D, respectively. The heart and lung are both associated with region A, with the heart typically corresponding to the tongue tip area. Experts analyze these regions for the presence of various TCM features, including spots, stasis, tooth-marks, rot, and dozens of other characteristics to determine the health status of each organ.

As illustrated in Figure 2, we present a series of examples depicting unhealthy organ conditions. In these images, Region A shows spots indicative of lung and heart issues. Region B exhibits tooth-marks, while Regions C and D display cracks, all of which are considered signs of potential health problems in TCM diagnosis. This comprehensive approach to data collection and annotation, guided by TCM principles, enables our deep learning models to learn from a rich set of visual cues and expert interpretations, forming a solid foundation for automated tongue diagnosis.

### 3.2 Annotation criteria for visceral conditions

The annotation of visceral conditions through tongue features follows a standardized assessment framework based on TCM principles. We categorize tongue features into three main groups as shown in Table 1: Body Features (examining color variations and surface characteristics), Fur Features (analyzing coating properties), and Other Features (including cracks and toothmarks). Each feature listed in Table 1 represents an unhealthy manifestation in TCM tongue diagnosis, along with its clinical description.

**Table 1:** Description of unhealthy tongue features used in TCM tongue diagnosis.

**Body features**	
Body pale	Light pink or pale colored tongue
Body red	Abnormally red tongue
Body deep red	Very deep red tongue
Body purple	Purple-tinged tongue
**Fur features**	
Fur thick	Thick coating
Fur yellow	Yellow coating
Fur black	Black or dark coating
Fur less	Thin coating
Fur none	No coating
Fur peeled	Partially or completely peeled coating
Fur moist	moist coating
Fur slippery	Excessive moisture in coating
Fur dry	Dry coating
Fur greasy	Thick, turbid coating
Fur rotten	Coating appears decomposed
**Other features**	
Crack	Fissures on tongue surface
Toothmark	Teeth impressions on sides of tongue
Spot	Small red or purple dots on tongue
Stasis	Dark purple or bluish patches

For region-specific diagnosis, these unhealthy features are evaluated differently across tongue regions. Table 2, derived from the authoritative textbook (Maciocia, 2015), illustrates which features are considered for each region in diagnosis, where Not Relevant (“NR”) indicates features that are either anatomically impossible, lack diagnostic significance for specific regions according to TCM principles, or represent extremely rare cases not encountered in our data collection. For instance, toothmarks present an interesting case: while they are anatomically visible along the lateral edges (traditionally associated with the liver region) and occasionally in the heart/lung area, their primary diagnostic significance indicates an underlying Spleen Qi Deficiency. This exemplifies how the physical location of tongue features may not always directly correspond to their diagnostic significance in TCM theory and clinical practice.

**Table 2:** Region-specific features and their clinical diagnostic significance in TCM.

**Features**	**Clinical diagnostic significance in TCM**	**Heart (tip)**	**Lung (front)**	**Spleen (center)**	**Liver (side)**	**Kidney (root)**
Body deep red	Severe heat or fire	Extreme heart fire	Heat toxicity in lung	Severe stomach fire/stomach heat toxicity	Severe liver fire/extreme liver yang rising	^*^NR
Spot	Blood heat or stasis	Heart fire	Lung heat	Stomach fire	Liver fire/heat	^*^NR
Fur thick	Dampness accumulation	Phlegm clouding Heart	Phlegm accumulation in lung	Food stagnation/stomach dampness	^*^NR	Kidney dampness
Fur yellow	Internal heat	Heart fire	Lung heat	Stomach heat	^*^NR	Kidney heat
Fur black	Severe internal cold or heat, critical conditions	^*^NR	^*^NR	Spleen Yang collapse with cold	^*^NR	Severe kidney yang collapse
Fur peeled	Damage to stomach yin	^*^NR	^*^NR	Severe stomach yin damage	^*^NR	Kidney essence insufficiency
Fur greasy	Dampness or phlegm accumulation	Phlegm misting Heart	Phlegm obstructing Lung	Dampness turbidity/food stagnation	^*^NR	Kidney phlegm-dampness
Fur rotten	Severe internal heat	Heart abscess	Lung abscess	Severe food stagnation	^*^NR	Severe endogenous heat
Toothmark	Qi deficiency or Dampness	^*^NR	^*^NR	Spleen Qi deficiency/with dampness	Liver Qi deficiency	Kidney Yang deficiency/with water retention

^*^NR for not relevant.

This structured annotation approach ensures consistency with TCM diagnostic principles while maintaining clinical practicality. The standardized criteria also facilitate reliable assessment across different practitioners and enable systematic analysis of tongue features in relation to visceral conditions.

To ensure annotation reliability, we implemented a rigorous three-person quality control mechanism. Two TCM practitioners (A and B) independently performed initial annotations following the standardized criteria outlined in Tables 1, 2. This standardized framework ensures consistency in feature assessment across different practitioners. For cases where practitioners A and B had divergent opinions, a senior TCM professor with extensive clinical experience reviewed the cases and made the final determination. Additionally, all disputed cases were thoroughly discussed among the three practitioners during regular review sessions to maintain diagnostic consistency and refine the annotation process.

This structured annotation approach, combined with our multi-level quality control process, ensures both consistency with TCM diagnostic principles and reliability of the dataset. The standardized criteria and verification mechanism facilitate reliable assessment across different practitioners and enable systematic analysis of tongue features in relation to visceral conditions. These defined features and their region-specific associations form the foundation for our subsequent feature extraction process.

### 3.3 Dataset structure

In our study, we adopted a specific evaluation protocol to facilitate comparisons with human performance. Our dataset, comprising a total of 4,645 tongue images, was divided into three subsets: 3,455 images for training (~75%), 295 images for validation (about 5%), and 895 images for testing (around 20%).

This split strategy was chosen to balance the need for a robust training set while maintaining a substantial test set for meaningful comparisons with human practitioners. Although we didn't use a five-fold cross-validation approach, which would have required extensive human labeling efforts, our fixed split provides a reliable basis for model evaluation and comparison with human performance.

Table 3 presents the distribution of unhealthy organ samples (labeled as 1) across our dataset splits for each of the five viscera considered in our study.

**Table 3:** Distribution of unhealthy organ samples (label = 1) across datasets.

**Viscera**	**Train**	**Val**	**Test**	**Total**
Heart	1,583	135	385	2,103
Lung	2,042	188	509	2,739
Spleen	3,435	292	889	4,616
Liver	2,174	202	562	2,938
Kidney	2,317	201	593	3,111

As shown in the table, there is a notable imbalance in the distribution of unhealthy samples across different organs. The spleen shows the highest number of unhealthy samples (4,616 in total), while the heart has the lowest (2,103 in total). This imbalance reflects the real-world distribution of visceral condition issues in our collected data, providing a realistic scenario for our model to learn from.

The consistent ratio of samples across train, validation, and test sets for each organ ensures that this distribution is maintained in all phases of our experiment. This consistency is crucial for reliable model evaluation and comparison with human performance.

It's worth noting that the numbers in the table represent only the unhealthy samples. The healthy samples (labeled as 0) for each organ would complement these figures to make up the total dataset size of 4,645 images.

This carefully structured dataset allows us to train our model on a diverse range of tongue images, validate its performance, and conduct a fair comparison with practitioners using a substantial test set.

## 4 Methodology

In this section, we first present the preliminary formulation of our framework for recognizing visceral condition from tongue images. We then describe our tongue organ region partitioning algorithm. Finally, we introduce our proposed OrganNet, which establishes a bridge between deep learning and real-world TCM diagnosis by simulating the actual diagnostic process.

### 4.1 Preliminary

Let *I*∈ℝ^*H*×*W*×3^ denote an input tongue image, where *H* and *W* represent the height and width of the image respectively. Our goal is to predict the health status of *N* organs, where *N* = 5 in our current setup, corresponding to the heart, liver, spleen, lung, and kidney. We define the set of visceral condition as $Y=y_{1},y_{2},...,y_{N}$, where *y*_*i*_∈0, 1 represents the health status of the *i*-th organ (0 for healthy, 1 for unhealthy).

Our framework incorporates a tongue organ region partitioning algorithm $P:I\to R_{1},R_{2},...,R_{N}$, where *R*_*i*_ represents the region of the tongue image corresponding to the *i*-th organ. This is followed by an OrganNet $F:\left(I,R_{1},R_{2},...,R_{N}\right)\to \hat{Y}$, where $\hat{Y}={ŷ}_{1},{ŷ}_{2},...,{ŷ}_{N}$ are the predicted health statuses for each organ.

### 4.2 Tongue organ region partitioning algorithm

**Figure 3:**
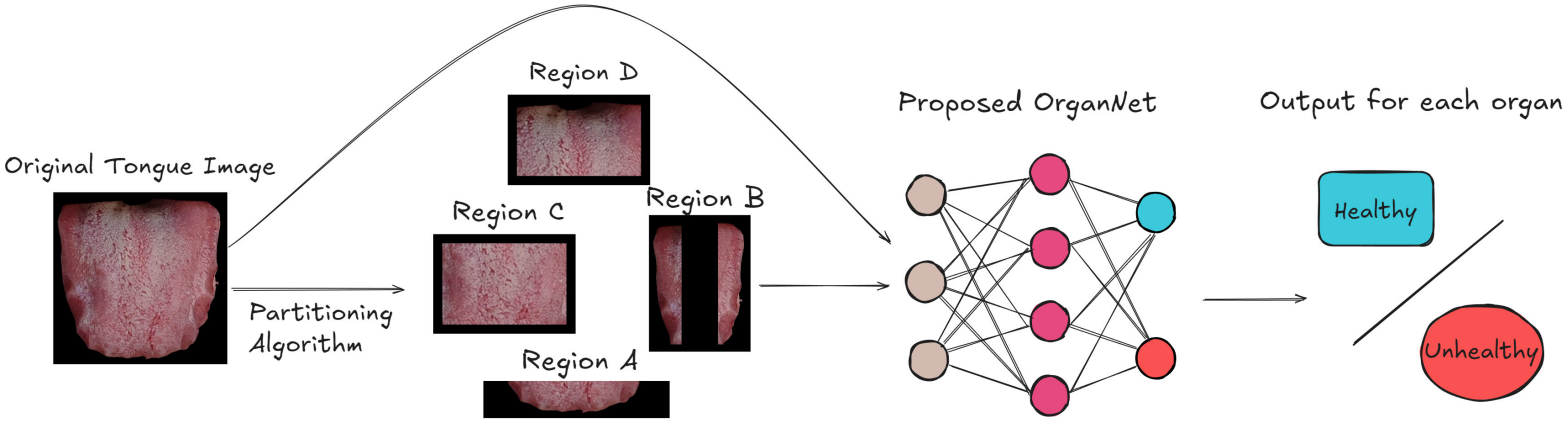
Overview of our framework for visceral condition recognition from tongue images. The tongue image is first partitioned into organ-specific regions. Both the original image and partitioned regions are then input to OrganNet, which generates health status predictions for multiple organs.

As shown in Figure 3, the first step in our approach is to input the tongue images into our Tongue Image Region Partitioning Algorithm. This algorithm follows the traditional TCM diagnostic principles documented in the comprehensive Diagnostics of Traditional Chinese Medicine textbook (Maciocia, 2015), and was validated through consultations with five qualified TCM practitioners (four licensed practitioners and one TCM professor with over 50 years of clinical experience), who confirmed that different tongue regions correspond to specific visceral conditions. Based on these principles, we designed our algorithm to divide each tongue image into different regions for subsequent analysis.

The algorithm, as detailed in Algorithm 1, takes a set of color tongue images (*X*_1_, *X*_2_, …, *X*_*n*_) along with their height (*h*) and width (*w*) as input. It then processes each image to partition it into four distinct regions: A, B, C, and D.

**Algorithm 1:**
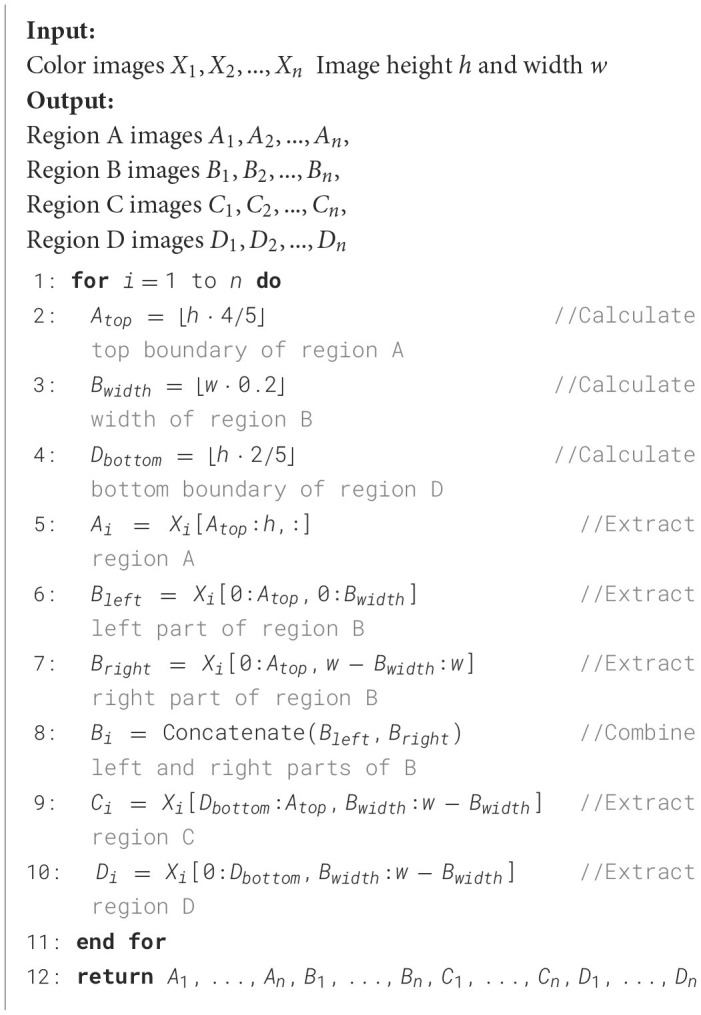
Tongue image region partitioning algorithm.

The partitioning process is as follows:

- Region A is defined as the bottom fifth of the image, representing the tip of the tongue.
- Region B consists of two vertical strips on the left and right sides of the image, each taking up 20% of the image width.
- Region C is the central area of the tongue, excluding the bottom fifth and top two-fifths of the image.
- Region D is the root of the tongue, represented by the top two-fifths of the central area.

The algorithm calculates the boundaries for these regions using proportions of the image dimensions. It then extracts each region from the original images, creating separate sets of images for each region (*A*_1_, …, *A*_*n*_, *B*_1_, …, *B*_*n*_, *C*_1_, …, *C*_*n*_, *D*_1_, …, *D*_*n*_).

This partitioning approach allows us to analyze different areas of the tongue separately, which is important because different regions of the tongue can provide different diagnostic information in traditional Chinese medicine.

### 4.3 OrganNet: bridging deep learning and TCM diagnosis

**Figure 4:**
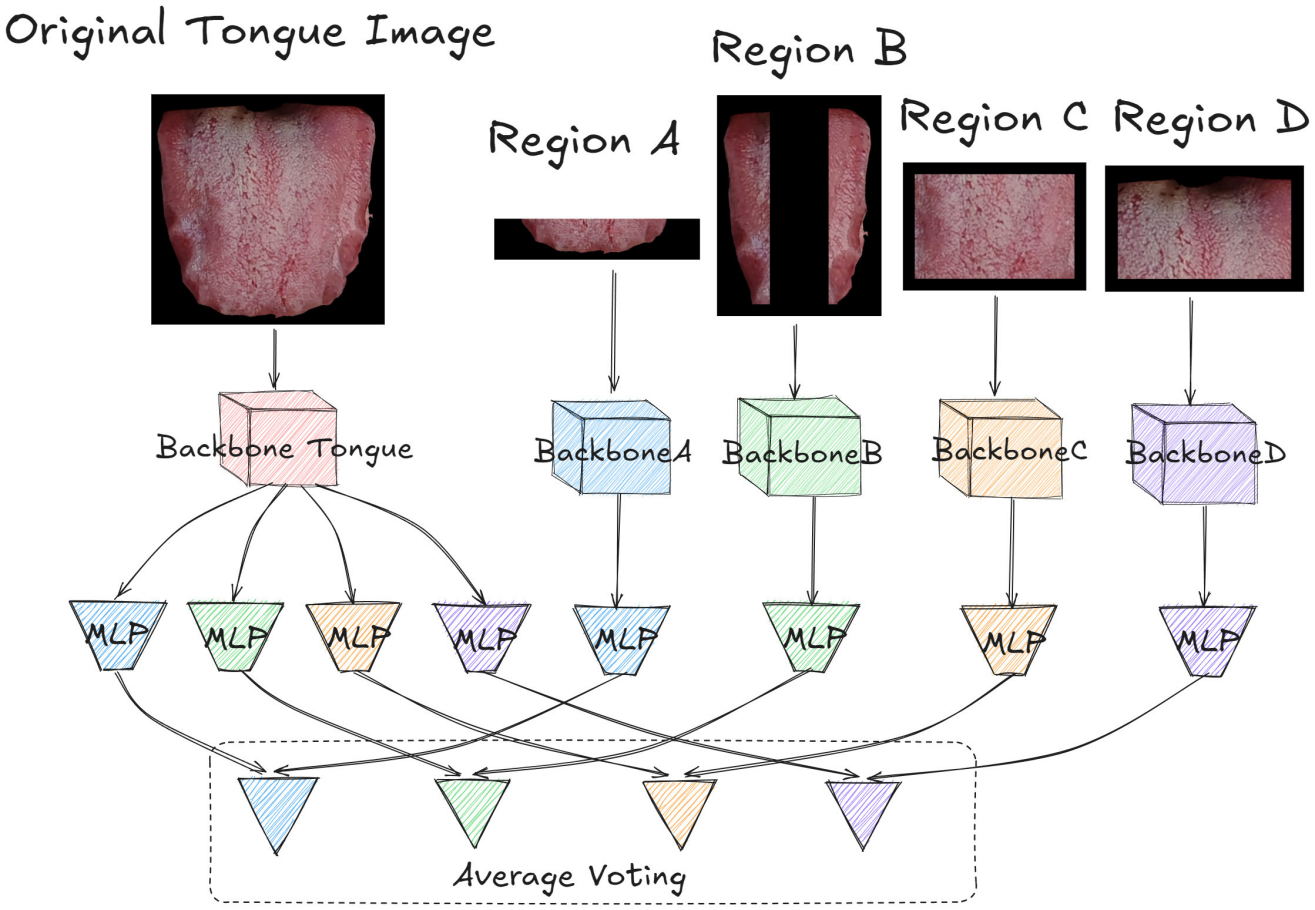
Detailed architecture of OrganNet. The original tongue image and partitioned regions are processed by separate backbones. The original image backbone predicts for all organs, while region-specific backbones predict for their corresponding organs. Final predictions are generated through average voting, combining global and local tongue features. Each block in the network has its own independent weights, with no weight sharing between blocks.

In our proposed OrganNet, the original tongue image is first partitioned into different regions. As shown in Figure 4, the original tongue image and the partitioned region images are input into different backbones of OrganNet for processing. The red backbone labeled “Tongue” represents the processing path for the original tongue image. The blue BackboneA processes Region A, the green BackboneB processes Region B, the yellow BackboneC processes Region C, and the purple BackboneD processes Region D. This design allows the network to capture both global and local features of the tongue image simultaneously.

After processing, the Backbone Tongue extracts the features of organs represented by the four regions. Therefore, it splits into four different MLPs, with different colors representing different regional tasks. The blue MLP corresponds to Region A and predicts heart and lung health, the green MLP corresponds to Region B and predicts liver health, the yellow MLP corresponds to Region C and predicts spleen health, and the purple MLP corresponds to Region D and predicts kidney health.

For the other region-specific backbones, each connects to an MLP of the corresponding color, specifically predicting the health status of the organs represented by that region. It's worth noting that the parameters between these MLPs and Backbone Tongue are not shared; each MLP independently learns features for its specific task.

Finally, the logits output from the MLPs of the Backbone Tongue are combined with the outputs from the corresponding region-specific MLPs through average voting, based on the organs they represent, to obtain the final prediction. This average voting process can be formalized as follows:

For each organ *i*, the final logit *L*_*i*_ is calculated as:


(1) $$\begin{array}{l} L_{i}=\frac{1}{N}\left(L_{i,full}+L_{i,region}\right), \end{array}$$

where *N* is the number of networks contributing to the prediction (in this case, *N* = 2), *L*_*i, full*_ is the logit from the full tongue image for organ *i* and *L*_*i, region*_ is the logit from the corresponding single region network for organ *i*. This formula applies to all organs: heart and lung (Region A), liver (Region B), spleen (Region C), and kidney (Region D). The final prediction for each organ is determined by whether *L*_*i*_ is >0.5. As shown in Figure 1, the blue (Region A) voting results represent heart and lung health status, the green (Region B) results represent liver health status, the yellow (Region C) results represent spleen health status, and the purple (Region D) results represent kidney health status.

The design of OrganNet effectively integrates global and local features of the tongue image, mimicking the TCM diagnostic process of examining both overall appearance and specific organ-related regions. By combining this holistic approach with deep learning, OrganNet potentially delivers more comprehensive and accurate diagnostic predictions for TCM.

### 4.4 Objective function

Given the nature of our tongue image analysis task, which involves five simultaneous binary classifications for different visceral condition, we adopted the Asymmetric Loss (Ridnik et al., 2021) for multi-label classification. This approach allows us to handle all visceral condition classifications concurrently while effectively addressing the class imbalance issue present in our dataset.

Our objective function is defined as:


(2) $$\begin{array}{l} L=\frac{1}{N}\overset{N}{\underset{j=1}{\sum }}\overset{5}{\underset{i=1}{\sum }}L_{ASL}\left(p_{ij},y_{ij}\right), \end{array}$$

where *N* is the total number of samples, *i* represents each of the five viscera, *y*_*ij*_ is the true label (0 or 1) for the *i*-th organ of the *j*-th sample, and *p*_*ij*_ is the predicted probability.

The Asymmetric Loss function *L*_*ASL*_ for each sample and organ is defined as:


(3) $$L_{ASL}(p,y)=\{\begin{array}{ll} {\left(1-p\right)}^{\gamma }\log(p) & \text{if }y=1\\ {\left(p\right)}^{\gamma }\log(1-p) & \text{if }y=0. \end{array}$$

Here, γ≥0 is a hyperparameter that controls the asymmetry of the loss function. When γ>0, the loss function puts more emphasis on hard examples (those with low prediction probability for their true class), which helps to address the class imbalance issue.

## 5 Experiments and results

In this section, we present our experimental setup and results, including quantitative comparisons with state-of-the-art methods and practitioners, visualization analysis, and ablation studies. It is important to note that our experiments involved human evaluation. Significantly, while the ground truth labels in our dataset were obtained through comprehensive on-site observations, the human evaluation in our experiments was based solely on single-view tongue images.

### 5.1 Experiment setup

#### 5.1.1 Implement details

In our implementation, we employed ResNet101 as the backbone network for its proven effectiveness in various computer vision tasks, offering a good balance between depth and computational efficiency. All experiments were conducted on a system equipped with an NVIDIA GeForce RTX 4090 GPU, which provides 24GB of GDDR6X memory, offering ample capacity for our model training and inference processes. Our implementation was based on the PyTorch framework, leveraging its dynamic computational graph and GPU acceleration capabilities. Prior to feeding the images into our model, we applied standard preprocessing techniques including resizing the images to 224 × 224 pixels and normalizing the pixel values. We trained our model using the AdamW optimizer with a learning rate of 0.0005 and a batch size of 64, which has shown good performance in various deep learning tasks. To improve the robustness of our model, we employed some common data augmentation techniques such as random horizontal flips.

#### 5.1.2 Evaluation metrics

To evaluate the performance of our tongue image analysis model, we employed two widely used metrics: accuracy and F1-score. Before delving into these metrics, we first define the following terms:

- True positive (TP): correctly identified unhealthy organ condition.
- True negative (TN): correctly identified healthy organ condition.
- False positive (FP): incorrectly identified unhealthy organ condition (Type I error).
- False negative (FN): incorrectly identified healthy organ condition (Type II error).

Accuracy Accuracy is defined as the ratio of correctly predicted observations to the total observations. It is calculated using the following formula:


(4) $$\begin{array}{l} \text{Accuracy}=\frac{\text{TP}+\text{TN}}{\text{TP}+\text{TN}+\text{FP}+\text{FN}}. \end{array}$$

F1-score: The F1-score is the harmonic mean of precision and recall, providing a balanced measure of the model's performance. It is particularly useful for imbalanced datasets. The F1-score is calculated as follows:


(5) $$\begin{array}{l} \text{F1-score}=2\times \frac{\text{Precision }\times \text{ Recall}}{\text{Precision }+\text{ Recall}}, \end{array}$$

where Precision and Recall are defined as:


(6) $$\begin{array}{l} \text{Precision}=\frac{\text{TP}}{\text{TP}+\text{FP}}, \end{array}$$

(7) $$\begin{array}{l} \text{Recall}=\frac{\text{TP}}{\text{TP}+\text{FN}}. \end{array}$$

These metrics provide a comprehensive assessment of our model's performance, considering both the overall accuracy and the balance between precision and recall, which is crucial for medical diagnosis tasks like visceral condition assessment through tongue image analysis.

### 5.2 Quantitative comparison with advanced models and human evaluation

Tables 4, 5 present a comprehensive comparison of different models and TCM experts in visceral condition assessment. It's important to note that the TCM expert results represent a consensus decision from three experienced practitioners. Due to the unavailability of specific model codes, differing task descriptions, or incompatible methodologies of tongue image analysis methods mentioned in related works section, direct comparisons were not feasible. Therefore, we selected several popular and advanced models for comparison with our proposed method. Our proposed OrganNet model demonstrates superior performance compared to these advanced baseline models across various metrics.

**Table 4:** Comparison of accuracy (%) for visceral condition assessment across different models and TCM experts.

**Method**	**Heart**	**Lung**	**Spleen**	**Liver**	**Kidney**	**Average**
ResNet101 (He et al., 2016)	71.17	65.36	99.33	77.21	77.21	78.06
EfficientNet (Tan, 2019)	72.96	68.27	99.33	78.44	77.88	79.37
ViT (Dosovitskiy, 2020)	71.62	63.58	99.33	65.70	66.37	73.32
SwinTransformer (Liu et al., 2021)	62.79	57.43	99.33	62.91	66.26	69.74
TCM experts	74.41	70.84	100.0	81.11	80.22	81.32
OrganNet (Ours)	73.41	72.29	99.78	80.78	80.45	81.34

**Table 5:** Comparison of F1-scores for visceral condition assessment across different models and TCM experts.

**Method**	**Heart**	**Lung**	**Spleen**	**Liver**	**Kidney**	**Average**
ResNet101 (He et al., 2016)	70.14	74.30	99.66	82.11	84.16	82.07
EfficientNet (Tan, 2019)	68.57	73.65	99.66	83.29	83.98	81.83
ViT (Dosovitskiy, 2020)	66.93	72.74	99.66	76.48	79.76	79.11
SwinTransformer (Liu et al., 2021)	51.67	70.58	99.66	77.20	79.70	75.76
TCM experts	70.90	75.81	100.0	85.90	85.41	83.61
OrganNet (Ours)	70.54	75.97	99.88	84.96	85.83	83.44

In terms of average accuracy (Table 4), OrganNet achieves the highest score of 81.34%, marginally surpassing TCM experts (81.32%) and significantly outperforming other deep learning models such as EfficientNet (79.37%), ResNet101 (78.06%), ViT (73.32%), and SwinTransformer (69.74%). Notably, OrganNet shows particular strength in lung assessment with an accuracy of 72.29%, compared to 70.84% for TCM experts and 68.27% for the next best model, EfficientNet.

The F1-score comparison (Table 5) reveals similar trends. OrganNet achieves an average F1-score of 83.44%, closely trailing TCM experts (83.61%) and outperforming other models including ResNet101 (82.07%), EfficientNet (81.83%), ViT (79.11%), and SwinTransformer (75.76%). OrganNet excels in kidney assessment with an F1-score of 85.83%, surpassing even TCM experts (85.41%).

It's worth noting that while TCM experts maintain a slight edge in overall performance, OrganNet outperforms them in specific organs, such as lung accuracy and kidney F1-score. This suggests that our model has not only reached a level comparable to human experts but may even surpass them in certain aspects.

These results underscore the effectiveness of our proposed method and its potential to significantly contribute to the automation and standardization of TCM tongue diagnosis. As we continue to refine and optimize OrganNet, we anticipate further improvements that could consistently exceed practitioner performance across all organs in the near future.

### 5.3 CAM visualization

To gain deeper insights into how our model makes decisions and to validate the effectiveness of our region-based approach, we employed Gradient-weighted Class Activation Mapping (Grad-CAM) (Selvaraju et al., 2017) for visualization. This technique allows us to highlight the areas of the image that are most influential in the model's decision-making process.

**Figure 5:**
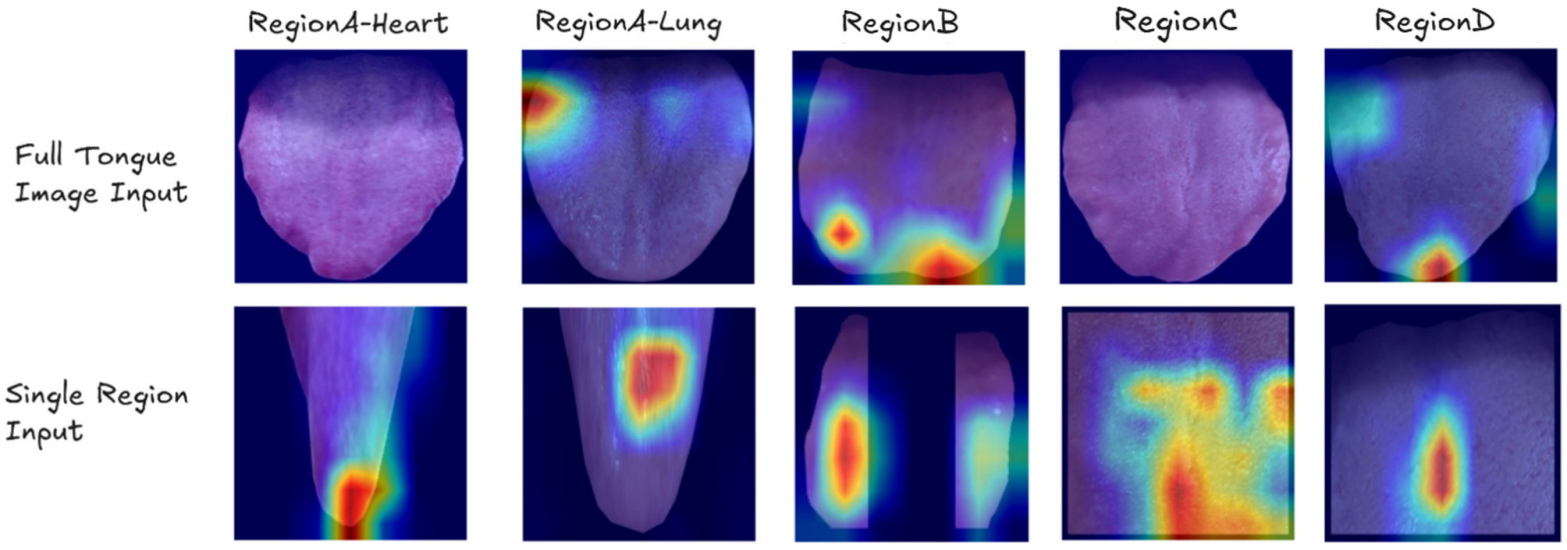
Examples of Class Activation Maps (CAMs) showing cases where ResNet fails when using the entire tongue image as input, but succeeds when using individual tongue regions. Each column represents images where the corresponding organ's label is 1. The CAMs highlight the areas of focus for the model, demonstrating that the model sometimes fails to identify the correct regions of interest when presented with the full tongue image, but accurately identifies the relevant areas when given specific tongue regions.

Figure 5 presents examples of Class Activation Maps (CAMs) for various visceral condition assessments. These visualizations compare the performance of ResNet when using the entire tongue image as input versus when using individual tongue regions. Each column in the figure represents images where the corresponding organ's health status is labeled as abnormal (label 1).

The CAMs clearly demonstrate that when presented with the full tongue image, the model often misses the correct regions of interest. However, when given specific tongue regions, it accurately identifies the relevant areas for each organ's health assessment. This visual evidence strongly supports the efficacy of our region-based approach in OrganNet.

For example, in heart assessment (Region A), the single-region model can detect subtle spots that are indicative of heart-related issues. These spots might be overlooked when the model analyzes the entire tongue image. Similarly, for spleen assessment (Region C) and kidney assessment (Region D), the region-specific models successfully identify cracks in the tongue surface, which are known to be associated with these organs' health in traditional Chinese medicine diagnosis. These fine details are often missed when the full tongue image is used as input, highlighting the advantage of our region-based approach in capturing organ-specific features.

### 5.4 Ablation study

To gain a deeper understanding of the effectiveness of our proposed OrganNet model and evaluate the contribution of its various components, we conducted a detailed ablation study. We compared the performance of individual region networks against the full OrganNet model for visceral condition assessment. To ensure a fair comparison, these single region networks also used ResNet101 as their backbone.

Tables 6, 7 present the accuracy and F1-scores, respectively, for different methods across the five viscera. As we can observe from these tables, the performance varies between ResNet101 and single region networks for different organs. For instance, in terms of accuracy (Table 6), ResNet101 outperforms Region A Net for heart assessment (71.17% vs. 65.03%), while Region A Net performs better for lung assessment (68.27% vs. 65.36%). Similarly, for F1-scores (Table 7), we see Region A Net excelling in lung assessment (77.10% vs 74.30% for ResNet101), while ResNet101 performs better for heart assessment.

**Table 6:** Ablation study: comparison of accuracy (%) for visceral condition assessment using individual region networks and full OrganNet.

**Method**	**Heart**	**Lung**	**Liver**	**Spleen**	**Kidney**
ResNet101	71.17	65.36	77.21	99.33	77.21
RegionA net	65.03	68.27	–	–	–
RegionB net	–	–	77.43	–	–
RegionC net	–	–	–	98.21	–
RegionD net	–	–	–	–	78.10
OrganNet (Ours)	73.41	72.29	80.78	99.78	80.45

**Table 7:** Ablation study: comparison of F1-scores for visceral condition assessment using individual region networks and full OrganNet.

**Method**	**Heart**	**Lung**	**Liver**	**Spleen**	**Kidney**
ResNet101	70.14	74.30	82.11	99.66	84.16
RegionA net	69.04	77.10	–	–	–
RegionB net	–	–	82.56	–	–
RegionC net	–	–	–	99.10	–
RegionD net	–	–	–	–	83.04
OrganNet (ours)	70.54	75.97	84.96	99.88	85.83

The final results demonstrate the effectiveness of this strategy. OrganNet consistently outperforms both ResNet101 and individual region networks across all organs in terms of both accuracy and F1-score. For instance, in liver assessment, OrganNet achieves an accuracy of 80.78% and an F1-score of 84.96%, surpassing both ResNet101 (77.21% accuracy, 82.11% F1-score) and Region B Net (77.43% accuracy, 82.56% F1-score).

These mixed results highlight the importance of both local and global features in tongue diagnosis. To strike an optimal balance between local and global information, we implemented our OrganNet using an average voting method, combining the strengths of both approaches.

## 6 Discussion

### 6.1 Dataset distribution and clinical reality

The distribution patterns in our dataset, particularly the high proportion of stomach/spleen cases, deserve special attention. This apparent imbalance reflects several key clinical realities in TCM practice. First, the stomach/stomach system, as the “postnatal foundation” in TCM theory, serves as the central hub for digestion and metabolism. Its dysfunction typically manifests clearly on the surface of the tongue, making it one of the most common conditions observed. Second, modern lifestyle factors, including irregular eating habits, emotional stress, and poor sleep patterns, directly impact this system. Third, the vulnerability of the stomach/spleen system to external pathogenic factors, especially in humid and hot climates, leads to frequent pathological conditions such as dampness and phlegm. Lastly, its interconnected nature with other organ systems makes it a sensitive indicator of overall health status.

Rather than viewing this distribution as a sampling bias, it represents the natural occurrence of tongue attributes in clinical practice. These samples, collected under real-world conditions, reflect authentic clinical scenarios. The diverse image quality in our dataset further enhances our model's generalization ability and clinical applicability. In the context of AI development, such “natural bias” serves as valuable prior knowledge, potentially enhancing model accuracy by aligning with real-world diagnostic patterns. To further improve representativeness, we plan to expand our dataset to include more samples across different ages, genders, regions, and disease types, while maintaining this clinically representative distribution. Our Data Collection section and Table 2 detail our comprehensive diagnostic process and annotation standards, ensuring data quality and standardization.

### 6.2 Imaging quality and environmental factors

In clinical deployments, maintaining consistent imaging quality presents a fundamental challenge for automated tongue diagnosis systems. Environmental factors such as lighting conditions can significantly impact image acquisition and subsequent analysis. While standardized imaging devices (Jiang et al., 2022) could effectively eliminate environmental interference factors when available, our neural network architecture can also help mitigate these impacts in settings where specialized equipment is not accessible. This flexibility in deployment options ensures our model's applicability across different clinical environments.

### 6.3 Complex organ relationships

The complexity of viscera-bowels relationships, particularly the interrelated conditions of the liver-gallbladder and spleen-stomach, is not fully captured by the current models. While our current approach employs foundational machine learning models chosen for their interpretability and clinical integration potential, we recognize that these basic models may not fully capture the sophisticated interdependencies in TCM diagnostics.

### 6.4 Other limitations and challenges

The limited diversity within the dataset may compromise the model's generalizability across different ethnicities and regions. Additionally, the variability in diagnostic interpretations among different TCM practitioners presents another significant challenge. A static image may not capture all the nuances that a TCM expert would observe in person, highlighting the inherent limitations of single-image analysis in tongue diagnosis.

### 6.5 Future research directions

To address these challenges and further improve our system's performance, several key areas require attention. A fundamental priority is bridging the gap between TCM practitioners and AI developers. To achieve this, we are developing a systematic diagnostic application that provides AI-assisted reference diagnoses to TCM practitioners. We plan to conduct randomized controlled trials comparing outcomes between TCM+AI-assisted diagnosis versus traditional TCM diagnosis alone to scientifically validate the system's clinical value.

Moreover, we will organize a large-scale multi-center clinical validation study addressing the variability in diagnostic interpretations among different TCM practitioners. Through careful stratified sampling, we will ensure our evaluation team includes junior, intermediate, and senior practitioners from various regions and diagnostic backgrounds, providing a comprehensive assessment of our system's performance across different expertise levels. This validation study will also inform our cross-cultural validation efforts through: (1) collaborating with experts from various traditional medicine systems to collect their diagnostic standards, (2) establishing a multilingual terminology mapping system to bridge different medical systems, and (3) analyzing regional disease patterns to optimize model adaptability.

On the technical front, we will focus on enhancing system capabilities through video-based approaches and more advanced AI architectures. We will collect and annotate video-format tongue datasets, implementing advanced architectures such as 3DCNN, temporal attention mechanisms, or TCN (Lea et al., 2017), while introducing graph neural networks to better represent the intricate relationships between organs and their mutual influences in TCM theory. These enhancements will enable our system to capture temporal information from complete video sequences rather than relying on single static images.

To ensure sustainable development, we are establishing a continuous optimization mechanism with regular dataset updates and algorithm refinements based on clinical feedback. An open research platform will facilitate broader participation from both TCM and AI communities. The integration of these enhancements will be crucial for capturing the full complexity of TCM diagnostic relationships, potentially enabling more accurate diagnoses and real-time monitoring of visceral conditions.

## 7 Conclusions

In this study, we have addressed a significant gap in AI-assisted Traditional Chinese Medicine (TCM) tongue diagnosis by focusing on the critical aspect of visceral condition assessment. We introduced a novel dataset linking tongue images with visceral condition, providing a valuable foundation for future research. Additionally, we proposed OrganNet, an innovative network designed to bridge the gap between TCM diagnosis and deep learning techniques, significantly reducing learning complexity and enhancing interpretability. Notably, our method has achieved performance levels comparable to those of experienced TCM practitioners. Despite our significant progress, challenges and limitations remain, including dataset imbalance, limitations of single-image analysis, variability in diagnostic interpretations, and modeling complex viscera-bowels relationships. To address these challenges, our future research will focus on developing video-based dynamic tongue analysis systems, expanding diverse datasets, implementing standardization protocols and adaptive learning techniques, and improving methods for modeling complex organ relationships. Through these efforts, we aim to further enhance the accuracy and practicality of our system, advancing the integration of AI-assisted diagnosis in TCM practice.

## Data Availability

The raw data supporting the conclusions of this article will be made available by the authors, without undue reservation.
